# Psychometric Properties and Validation of the Polish Version of the 12-Item World Health Organization Disability Assessment Schedule 2.0 in Patients with Huntington’s Disease

**DOI:** 10.3390/jcm10051053

**Published:** 2021-03-04

**Authors:** Agnieszka Ćwirlej-Sozańska, Bernard Sozański, Mateusz Kupczyk, Justyna Leszczak, Andrzej Kwolek, Anna Wilmowska-Pietruszyńska, Agnieszka Wiśniowska-Szurlej

**Affiliations:** 1 Institute of Health Sciences, Medical College of Rzeszow University, 35-310 Rzeszow, Poland; mateusz-kupczyk@wp.pl (M.K.); justyna216@op.pl (J.L.); kwoleka@o2.pl (A.K.); wisniowska@vp.pl (A.W.-S.); 2 Institute of Medicine, Medical College of Rzeszow University, 35-310 Rzeszow, Poland; benieks@poczta.onet.pl; 3 Faculty of Medicine, Lazarski University, 02-662 Warsaw, Poland; anna.wilmowska@gmail.com

**Keywords:** ICF, disability, neuropsychological assessment

## Abstract

Background: Huntington’s disease is a progressive neurodegenerative disorder that usually manifests in adulthood and is inherited in an autosomal dominant manner. The main aim of the study was to assess the psychometric properties of the 12-item WHO Disability Assessment Schedule (WHODAS) 2.0 in studying the level of disability in people with Huntington’s disease. Method: This is a cross-sectional study that covered 128 people with Huntington’s disease living in Poland. We examined scale score reliability, internal consistency, convergent validity, and known-group validity. The disability and quality of life of people with Huntington’s disease were also assessed. Results: The scale score reliability of the entire tool for the research group was high. The Cronbach’s α test result for the whole scale was 0.97. Cronbach’s α for individual domains ranged from 0.95 to 0.79. Time consistency for the overall result was 0.99 and for particular domains ranged from 0.91 to 0.99, which confirmed that the scale was consistent over time. All of the 12-item WHODAS 2.0 domains negatively correlated with all of the Huntington Quality of Life Instrument (H-QoL-I) domains. All correlation coefficients were statistically significant at the level of *p* < 0.001. The results obtained in the linear regression model showed that with each subsequent point of decrease in BMI the level of disability increases by an average of 0.83 points on the 12-item WHODAS 2.0 scale. With each subsequent year of the disease, the level of disability increases by an average of 1.39 points. Conclusions: This is the first study assessing disability by means of the WHODAS 2.0 in the HD patient population in Poland, and it is also one of the few studies evaluating the validity of the WHODAS 2.0 scale in assessing the disability of people with HD in accordance with the recommendations of DSM-5 (R). We have confirmed that the 12-item WHODAS 2.0 is an effective tool for assessing disability and changes in functioning among people with Huntington’s disease.

## 1. Introduction

Huntington’s disease (HD) is a progressive neurodegenerative disorder that usually manifests in adulthood and is inherited in an autosomal dominant manner. The motor symptoms of the disease include chorea, dyskinesia, and dystonia. Huntington’s disease is accompanied by psychiatric disorders and cognitive impairment, usually preceded by depression, anxiety, and sleep disorders [1].

The impairment of both motor and cognitive functions is a cause of disability in patients with HD [2]. A progressive movement disorder is the main source of functional disability in HD [3]. Cognitive impairment of patients with HD is primarily characterized by executive dysfunction, but also affects learning, memory, and planning. Cognitive decline is characteristic [4]. Emotional manifestations of HD also have an extremely negative impact on social and professional functioning. Most often they include apathy, irritability, and depression [5].

Unfortunately, with the passage of time the disease progresses, bringing with it an exacerbation of movement disorders, deteriorating cognitive functions and behavioral difficulties, which lead to further disability and dependence [6]. People with HD become completely dependent on other people over time [7].

Movement disorders in patients with HD significantly affect and differentiate their level of functioning [2], as motor and cognitive performance deteriorate over time. The decrease in functional performance significantly correlates with a decrease in quality of life (QoL) [8]. Studying the functional state and level of disability as well as the quality of life of patients with HD is recommended in order to assess the progress of the disease, implement interventions, monitor the patient’s condition, and optimize care [6]. Additionally, Power et al., in a review of the research, concluded that the impact of personal and environmental factors on the activity and participation of people with HD were poorly studied. The authors emphasized the great potential of the International Classification of Functioning, Disability, and Health (ICF) to assess the activity and participation of people with HD [9]. The assessment of a person’s functioning in the context of their health state and environmental factors is possible using the ICF and tools based on it [10]. The WHO Disability Assessment Schedule 2.0 (WHODAS 2.0) (assessing functioning and disability) is one such tool. The questionnaire is based on ICF categories related to activities and participation [11]. Downing et al. suggest that the WHODAS 2.0 can identify baseline and longitudinal differences in HD and may be useful in the functional assessment of a patient with HD as well as in clinical trials [12].

Due to the fact that the use of the WHODAS 2.0 to assess the disability of people with HD has not yet been thoroughly investigated anywhere, and there are no such studies conducted in Poland, the main objective of our work was to assess the psychometric properties and usefulness of the 12-item WHODAS 2.0 for the assessment of the level of functioning and disability of this group of patients. In addition, the disability and quality of life of people with Huntington’s disease were assessed, and the impact of selected factors on these parameters was analyzed.

## 2. Materials and Methods

### 2.1. Study Design and Participations

This is a cross-sectional study that covered people with Huntington’s disease living in Poland. The study was conducted in the period from 2018 to 2019. Six centers belonging to the European Huntington’s Disease Network (of which 3 agreed to conduct the research) and the Polish Huntington’s Disease Association were invited to participate in the study. Finally, the survey included 128 people diagnosed with Huntington’s disease. Ultimately, 115 complete interviews were analyzed (9 people left the study during its implementation, 4 questionnaires were incomplete, and the number of deficiencies could not be supplemented using statistical methods).

### 2.2. Procedures

The study was conducted by the use of a direct interview carried out by healthcare professionals properly trained in the use of the 12-item WHODAS 2.0. In the case of patients in the advanced stage of the disease, they were accompanied by their caregivers. Each patient qualified for the study was informed about its goals and gave their informed consent to participate in the study. If the disease was significantly advanced (IV according to the Total Functional Capacity Scale), the consent and the presence of the patient’s caregiver were also required. The caregiver’s task was to help in interviewing the subject. The following inclusion criteria were adopted: diagnosed Huntington’s disease, age over 18 years and informed consent to participate in the study.

### 2.3. Ethics

In accordance with the Helsinki Declaration, study participants were informed about the purpose and course of the study and they agreed to participate in it. Moreover, the consent of the Bioethics Committee at the University of Rzeszow was obtained for the implementation of this research (resolution annex No. 27/6/2017).

### 2.4. Outcome Measures

The research tools was the WHO Disability Assessment Schedule 2.0 (WHODAS 2.0 12-item version). Commonly used and validated instruments were selected as reference tools: the Huntington Quality of Life Instrument (H-QoL-I) and the Total Functional Capacity (TFC). In addition, the following data regarding the subject patients were analyzed: age, sex, place of residence, education, marital status, professional status, comorbid chronic diseases, BMI, and adaptation of the home interior and the environment to the needs of the subjects.

#### 2.4.1. The 12-item WHODAS 2.0

The WHODAS 2.0 has been developed on the basis of a comprehensive set of categories included in the International Classification of Functioning, Disability, and Health (ICF). The questionnaire is used to measure functioning, activity and participation in everyday life in the last 30 days. It measures the level of functional disability.

The 12-item version of WHODAS 2.0 contains two questions each from six areas of life:

Domain 1: Cognitive functions—understanding and communicating (questions 3 and 6)

Domain 2: Mobility—getting around (questions 1 and 7)

Domain 3: Self-care—taking care of hygiene, dressing, eating, and staying alone (questions 8 and 9)

Domain 4: Getting along—interacting with other people (questions 10 and 11)

Domain 5: Life activity—domestic responsibilities, leisure time, work, and school (questions 12 and 2)

Domain 6: Participation—joining in community activities, participation in social life (questions 4 and 5).

Answers to the questions were classified according to a five-point scale indicating the level of difficulty or problem: none (1 point), mild (2 points), moderate (3 points), severe (4 points), and extreme or inability to perform (5 points). A simple method of calculating the results was used [13]. The total score for global disability was therefore between 12 (no disability) and 60 (complete disability), with higher scores indicating a higher level of disability. According to WHO guidelines, the severity of functional disability is based on the calculated percentage as follows: none (0–4%), mild (5–24%), moderate (25–49%), severe (50–95%), and complete (96–100) [14].

In addition, individual domain results were also calculated, adding the results of two items in each domain [15].

When only one item from the 12 items of WHODAS 2.0 was missing, the missing item was assigned the average of the remaining 11 items (there were 4 such instances in the entire group). If more than one item was missing, no data was assigned and the questionnaire was rejected (2 questionnaires were rejected for this reason) [14].

#### 2.4.2. The Huntington Quality of Life Instrument (H-QoL-I)

The Huntington Quality of Life Instrument is a specific instrument for assessing the quality of life of people with Huntington’s disease. It consists of 11 questions, which are divided into 3 areas: motor functioning, psychological aspect, and social aspect. The answers to the questions were ranked according to a Likert scale. The answer can be chosen from 5 options depending on the frequency (very rarely or never, rarely, sometimes, often, very often, or always) or intensity (not at all, a little, medium, a lot, extremely). The calculations were carried out in accordance with the H-QoL-I User Guide. According to the rules, missing data from a given area were calculated as average responses to other elements of the same area (there were 3 such instances in the entire group). If more than one element was missing, the dimension result was considered missing and could not be calculated (2 questionnaires were rejected for this reason). The results are presented on a scale from 0 (representing the worst state) to 100 (the best state) [16,17].

#### 2.4.3. The Total Functional Capacity (TFC)

The severity of Huntington’s disease was assessed using a standardized the Total Functional Capacity (TFC) Scale. This scale is recommended by the Huntington’s Disease Workgroup of Promoting Excellence in End-of-Life Care and is one of the most frequently used measures in HD research [18]. The TFC determines the level of functioning by means of 5 questions in the areas of work, finance, housework, daily activities, and care requirements. Each question has three or four categories of answers (from 0 to either 2 or 3) (e.g., “occupation: 0 = unable, 1 = marginal work only, 2 = reduced capacity for usual job, 3 = normal”). The TFC ranges from 0 (total dependence) to 13 (normal) [19].

### 2.5. Statistical Analysis

Obtained data were analyzed using the STATISTICA version 12 software. Initial analyses of sociodemographic and health-related data were performed using descriptive statistics. The normal distribution of variables was tested using the Shapiro–Wilk test.

The first part of the results was devoted to assessing the validity of the 12-item WHODAS 2.0 regarding the disability assessment of people with Huntington’s disease by means of a psychometric tool. With regard to scale score reliability, internal consistency reliability was studied. Cronbach’s alpha was calculated, and as a critical cut-off value 0.70 was considered the minimum acceptable reliability [20]. Moreover, correlations between summary results, domains and items were also calculated. The minimum level of correlation was assumed to be a correlation of at least 0.6 (Klein’s criterion) [21]. A re-test was carried out in a group of 20 people. The average time between two measurements was 5 days (4–6 days). The reliability of the test-retest was analyzed using the Wilcoxon test (due to the lack of normal distribution) and an interclass correlation coefficient (ICC) [22]. Floor and ceiling effects were calculated by determining the percentage of participants who had the lowest or highest possible results for individual items of the 12-item WHODAS 2.0.

Pearson correlations were used to assess convergent validity (when pairs of variables were normally distributed) or Spearman correlations (when at least one variable was not normal) in order to determine the relationship between the WHODAS 2.0 and the H-QoL-I [23]. Correlations less than 0.3 were considered weak, 0.3–0.6 sufficient, and 0.6 or more were regarded as good or very good evidence of convergent validity [24]. Based on scientific research, it has been hypothesized that patients with a higher level of disability have a lower quality of life [25]. We also conducted an analysis of the known-group validity. In order to assess whether the questionnaire differentiates people with different health status, we checked whether it differentiates the disability of people with different stages of disease (Stage I–II and Stage III–IV). We hypothesized that patients with higher levels of disease have higher levels of disability [26].

With reference to the second part of the results, we discussed the disability and quality of life in the study group, and we implemented linear regression models to study which of the selected sociodemographic variables have a significant impact on the level of disability and quality of life of patients with Huntington’s disease, and to what extent. The quality of the models was assessed using the coefficient of determination R2. The level of statistical significance was assumed *p* < 0.05.

## 3. Results

### 3.1. Sociodemographic and Health-Related Data of the Study Group

Sociodemographic and general health characteristics are presented in Table 1. The average age of the people surveyed was 45.59 years. Most of the subjects were women, people living in urban areas, living with a spouse or partner, with secondary education or higher and not working because of their health status. The average duration of the disease in the group since it was diagnosed was 6.74 years (SD 4.41). Most of the respondents were diagnosed with at least one comorbid disease (77.39%). Most of the patients suffered from depression diagnosed by a doctor (78.26%). Additionally, the most common coexisting chronic diseases in the study group were hypertension (18.26%) and lumbar pain in the L-S segment (23.48%) or in the C segment (13.04%).

A significant average level of disability measured by the WHODAS 2.0 was found in the study group (Mean = 36.54; SD = 15.45). The biggest restrictions were found in the following areas: participation in social life, mobility, and life activities. With reference to the quality of life of the subjects measured by means of the H-QoL-I, it was at a medium level, with the highest score in the social domain and the lowest one in the psychological area.

### 3.2. Scale Score Reliability

#### 3.2.1. Internal Consistency Reliability

The scale score reliability of the entire 12-item WHODAS 2.0 for the research group was high. The Cronbach’s α test result for the whole scale was 0.97. Cronbach’s α for individual domains ranged from 0.95 to 0.79 (Table 2).

#### 3.2.2. Correlation Matrix between Summary Result, Domains and Items

In order to assess the choice of domains, the correlation matrix between the summary result of the Polish version of the 12-item WHODAS 2.0 and six domains was assessed. In all cases, the correlation was very high or high. Particularly high correlations are visible between the summary result and domains assessing life activities (r = 0.95), mobility (r = 0.94), or cognition (r = 0.94) (Table S1). Very high, statistically significant correlations were found between questions in a given domain and the overall result of WHODAS 2.0 (Table S2).

#### 3.2.3. Test-Retest

When assessing the significance of changes at the level of the scale in the retest study in relation to the test study, no significant differences were noted for any of the domains. The reliability of the test–retest method was confirmed by the ICC. Time consistency for the overall result was 0.99 and for particular domains ranged from 0.91 to 0.99, which confirmed that the scale was consistent over time (Table 2).

### 3.3. Floor and Ceiling Effects

The floor effect (an answer of “no problem”) value was from 6.96% (for item 5) to 32.17% (for item 11), while the ceiling effect (an answer of “extremely large” or “cannot do”) was found from 11.30% (for item 11) to 31.30% (for item 12).

### 3.4. Validity

#### 3.4.1. Convergent Validity

The convergent validity was tested by correlating the results obtained with the 12-item WHODAS 2.0 test and the results of the H-QoL-I questionnaire. All of the 12-item WHODAS 2.0 domains negatively correlated with all H-QoL-I domains, so the higher the WHODAS 2.0 score (higher disability), the lower the H-QoL-I score (lower quality of life). All correlation coefficients were statistically significant at the level of *p* < 0.001. All correlations were higher than 0.6, which would indicate at least good convergence (Table 3).

#### 3.4.2. Known-Group Validity

The Mann–Whitney test was used to assess the known-group validity of the 12-item WHODAS 2.0 questionnaire using an external criterion. We found significant differences between patients with different levels of disease advancement (Stage I–IV) in the levels of disability measured by the WHODAS 2.0. We have confirmed the hypothesis we put forward that adults with higher levels of advanced disease are characterized by a higher level of disability. The WHODAS 2.0 results for subjects with less and more advanced disease was significant (Table 4).

### 3.5. Linear Regression Model

#### 3.5.1. Impact of Different Factors on Disability

The relationship between selected factors and the disability of people with HD is shown in Table 5. With reference to the model assessing the impact of selected factors on disability, R2 was 64.53%, which indicates an acceptable model fit. The results obtained in the linear regression model show that with each subsequent point of decrease in BMI the level of disability increases by an average of 0.83 points on the 12-item WHODAS 2.0 scale. With each subsequent year of the disease, the level of disability increases by an average of 1.39 points. People with HD living in urban areas have, on average, 2.78 points higher disability than those living in rural areas. On average, professionally inactive patients have a higher disability level of 7.78 points than professionally active patients.

#### 3.5.2. Impact of Different Factors on Quality of Life

The relationship between selected factors and the quality of life of people with HD is shown in Table 6. With reference to the model assessing the impact of selected factors on quality of life, R2 was 63.20%, which indicates an acceptable model fit. The results obtained in the linear regression model indicate that with each subsequent point of decrease in BMI the level of quality of life decreases by an average of 1.46 points on the H-QoL-I scale. With each subsequent year of the disease, the quality of life rating decreases by an average of 2.76 points. People with HD living in urban areas have, on average, 4.05 points lower quality of life than those living in rural areas. On average, professionally inactive patients have a quality of life 9.70 points lower than professionally active subjects (Table 6).

## 4. Discussion

In our study, we have assessed the disability and quality of life of people with Huntington’s disease in Poland and the impact of selected factors on their functioning and quality of life. The 12-item WHODAS 2.0 questionnaire was used to assess disability. The American Psychiatric Association DSM-5 recommends the WHODAS 2.0 in the Diagnostic and Statistical Manual of Mental Disorders (DSM-5 (R)) for use in neuropsychiatric disorders. However, due to the fact that this questionnaire is not specific for Huntington’s disease and has never been used with patients in Poland, before interpreting the results, its psychometric properties for disability assessment were analyzed in this group of patients. We confirmed the reliability and validity of WHODAS 2.0 for testing people with HD. We noticed very good results of internal consistency reliability of this tool. The Cronbach’s α test result for the whole scale was 0.97, while for individual domains it ranged from 0.95 to 0.79. We found very high correlations between individual domains and the overall result as well as between individual questions and the overall score of the tool. Carlozzi et al., who validated the 12-item WHODAS 2.0 questionnaire in a group of 447 patients with Huntington’s disease, also obtained a high value of Cronbach’s α (0.94) [27]. Other researchers also confirmed the high internal consistency reliability of the 12-item WHODAS 2.0 in the assessment of patients with other diseases. Younus et al. while examining patients with Kashin–Beck disease obtained values of Cronbach’s α for individual domains in the range from 0.70 to 0.91 [28]. Axelsson et al. found values for Cronbach’s α in the 0.83–0.92 range in a study of patients with anxiety and stress disorders [29]. Schiavolin et al. obtained 0.88 for the value of Cronbach’s α when studying neurological patients [30].

With reference to our study, we confirmed the good repeatability of the 12-item WHODAS 2.0 in the study of people with HD. In the retest study compared to the test study, no significant differences were found in any of the analyzed domains. The ICC value for the overall result was 0.99, and for individual domains from 0.91 to 0.99. Younus et al. also confirmed the very good test–retest reliability of the 12-item WHODAS 2.0 [28], likewise Moreira et al. (ICC 0.77) [31] and Marom et al. (ICC 0.88) [32].

Regarding our study, the floor effect value for individual questions was from 6.96% to 32.17%, while a ceiling effect was found from 11.30% to 31.30%. Carlozzi et al. in their study showed a ceiling effect for 19.5% of participants, and they did not find any floor effects [27]. Differences in the results between our research and the ones presented by Carlozzi et al. may be due to the fact that we assessed the ceiling and floor effect for each question, while the aforementioned authors did so for the whole scale. Secondly, in our study the structure of the group was evenly distributed in terms of disease stage (Stage I–IV); it included people in the first stage of the disease as well as in the most severe stage.

In our study, the convergent validity was tested by correlating the results obtained with the 12-item WHODAS 2.0 test and the results of the H-QoL-I questionnaire. All correlations were negative, higher than 0.6 and were statistically significant (*p* < 0.001) which would indicate at least good convergence. Good convergent validity of this tool has also been confirmed by other authors. Carlozzi et al. found moderate significant correlations (−0.41 to −0.76) with other general measures of health-related quality of life (HRQoL) [27]. Tazaki et al. noted significant correlations with the WHOQOL-BREF results (*p* < 0.001) [33]. Luciano et al. confirmed the consistency of the 12-item WHODAS 2.0 with the results of the EuroQoL-5D (EQ-5D) questionnaire [34]. Schiavolin et al. found the convergence of the 12-item WHODAS 2.0, obtaining moderate correlations with instruments for assessing quality of life [30].

We confirmed that 12-item WHODAS 2.0 had satisfactory validity for people with various levels of disease. We found significant differences (*p* < 0.001) between patients with different levels of disease advancement (Stage I–IV) and the levels of disability measured by the WHODAS 2.0. We have confirmed the hypothesis we put forward that adults with higher levels of advanced disease are characterized by a higher level of disability. Carlozzi et al. also noted significant differences in disability measured by the use of WHODAS 2.0 in patients with milder and more severe forms of Huntington’s disease [27]. The ability of the 12-item WHODAS 2.0 to differentiate between various health conditions has also been confirmed by Schiavolin et al. [30].

In the group of examined patients with HD, we noticed a significant average level of disability measured by the 12-item WHODAS 2.0 (Mean = 36.54; SD = 15.45). The biggest restrictions occurred in participation in social life (Mean = 6.63; SD = 2.39), mobility (Mean = 6.48; SD = 2.78), and life activities (Mean = 6.47; SD = 2.90). Downing et al. assessed the disability of patients with HD by means of the 36-item WHODAS 2.0, similarly noting a high level of disability in the studied groups. In longitudinal studies they also found greater sensitivity of the WHODAS 2.0 than the TFC in recognizing functional changes in patients as the disease progresses [12]. Moreover, the results obtained by Kim et al. suggested that the 12-item WHODAS 2.0 can detect basic and longitudinal differences in prodromal HD and may be useful in clinical trials regarding HD, more so than the 36-item WHODAS 2.0 or the TFC [35].

Based on the applied linear regression model, we have selected factors that have a special impact on the disability of patients with HD. We have found that with each successive year of the disease the level of disability increases by an average of 1.39 points. Bylsma et al. demonstrated that the duration of the disease was one of the best predictors of functional disability measured by the use of the Huntington’s Disease Activities of Daily Living (HD-ADL) scale [36]. In addition, we have also observed that with each subsequent point of decrease in BMI the level of disability increases by an average of 0.83 points on the 12-item WHODAS 2.0 scale. Trejo et al. noted in patients with HD significant correlations between the total score of motor disability and BMI and arm circumference [37]. Van der Burg et al. found that lower BMI is associated with a higher rate of disease progression and may be an important predictor of disease progression [38]. Adequate nutrition management is very important in the prevention of low BMI and the significantly deteriorating functional capacity associated with it [39,40]. In our study, people with HD living in urban areas had, on average, 2.78 points higher disability than those living in rural areas. Perhaps this result is associated with a greater level of physical activity of people living in Polish rural areas, the possibility of free mobility and the need to perform various physical tasks in their household. Wallace et al. found that a higher level of physical activity positively correlates with lower cognitive and motor deficits, improving cognitive and daily functions and probably also motor functions in people in the prodromal and early stages [41]. Trembath et al. observed that an active lifestyle and environmental factors preventing passivity contribute to the later onset of HD symptoms and can have a positive effect on the course of the disease [42]. In the group of HD patients that we studied, the professionally inactive patients had an average disability level of 7.78 points higher than the professionally active ones. Beglinger et al. stated in their study that a decline in professional activity was common in people with HD, with 65.1% reporting some loss of ability to perform typical work, measured by means of the Unified Huntington’s Disease Rating Scale (UHDRS) and the TFC [43]. Vaccarino et al. noticed that people with HD, especially at an early stage, reported that they could continue to work professionally in their work environment if the workplace could be adapted to their needs [44]. Identifying factors that may facilitate or hinder the performance of work would thus increase the ability to modify the workplace, which could enable people with pre-HD to maintain employment for as long as they want and are able to work, and thus extend the time of self-reliance and independence [45].

In our study, the deepening of disability as the disease progressed was significantly associated with a decrease in quality of life. An increase in functional impairment significantly correlates with a decrease in QoL [8,46]. As for the group of people with HD studied by us, the quality of life of the subjects measured by the H-QoL-I was at a medium level (Mean = 52.69; SD = 26.67), with the highest component in the social domain (Mean = 69.86; SD = 26.85) and the lowest in the psychological domain (Mean = 42.39; SD = 28.12). Dorey et al., while examining quality of life using the H-QoL-I among 55 patients with HD, reported a higher overall quality of life (66.37). As in our own study, they found the highest quality of life in the social domain, but it was definitely higher than in our research (80.33). The lowest quality of life was found by Dorey et al. in the physical domain (56.96) and comparable in the psychological domain (58.06) [25]. Generally, these ratings were higher than in our study.

Based on the linear regression model used in our research, we have selected factors that have a special impact on the quality of life of patients with HD. They are the same as the factors determining disability in patients with HD. This is confirmed by the fact that quality of life is closely related to physical and mental health [47]. Ho et al. while examining the determinants of health-related quality of life stated that greater impairment of the HRQoL was associated with higher levels of depressed mood and lower functional capacity [48].

Similarly to the model examining the determinants of disability, we have found that with each successive year of the disease, the assessment of quality of life decreases by an average of 2.76 points. Emotional state is significantly related to the level of disability and the pace of progression of functional disorders in the course of the disease. Worsening emotional state and deterioration of physical condition reduce the social involvement of people with HD [45]. We have also noticed that with each subsequent point of decrease in BMI, quality of life decreases by an average of 1.46 points on the H-QoL-I scale. Since we have proved that the decrease in BMI is closely related to the progression of the disease and the progression of disability, this relationship seems to be obvious. People with HD living in urban areas represented, on average, 4.05 points lower quality of life than those living in rural areas. This is an interesting discovery. Our observations show that this may be related to the more frequent isolation of people suffering from Huntington’s disease in urban areas in a small living space and limited contact with the external environment, to a greater extent than in the case of people living in rural areas, where there is a possibility of free activity over the area of the entire homestead. The rural environment is also characterized by less social variability than the urban environment. The quality of life and disability of patients with HD has not thus far been compared with reference to the place where they live. This indicates an interesting direction for future research. On average, the professionally inactive patients had a quality of life 9.70 points lower than the professionally active ones. As with the factors discussed above, professional activity and capability decrease as the disease progresses. In addition, a relationship has been demonstrated between inactivity and lower quality of life [45].

Support for the mental and physical health of people with HD, as well as effective prevention and treatment of depression and anxiety, can be strategically targeted to help people with HD to perform their tasks and continue to be independent for as long as possible [45].

### Limitations

The limitation of the research is the cross-sectional measurement of the study, which excludes longitudinal observation of changes. Further studies should also be carried out in a larger group of patients and it is necessary to take into account the impact of various sociodemographic and environmental factors more specifically on disability and quality of life in patients with HD.

## 5. Conclusions

This is the first study assessing disability by means of the WHODAS 2.0 in the HD patient population in Poland, and it is also one of the few studies evaluating the validity of the WHODAS 2.0 scale in assessing the disability of people with HD in accordance with the recommendations of DSM-5 (R). We have confirmed that 12-item WHODAS 2.0 is an effective tool for assessing disability and changes in functioning of people with Huntington’s disease. Our research is one of the few studies determining the impact of various factors on the level of disability and quality of life of patients with HD. Although this issue is extremely convoluted, owing to the complexity and changing nature of this disease, it is very important due to the lack of causal treatment. Our work is an important contribution to future research in terms of analysis of the sociodemographic and environmental factors affecting the level of disability and quality of life of people with Huntington’s disease. 

## Figures and Tables

**Table 1:** General sociodemographic characteristics of the studied population.

Sociodemographic Characteristics (*n* = 115)	Number (*n*)/Mean	Percentage (%)/SD
**1. Age**	45.59	13.27
**2. Gender**		
female	62	53.91
male	53	46.09
**3. Place of residence**		
urban	61	53.04
rural	54	46.96
**4. Marital status**		
single	21	18.26
married or with a partner	78	67.83
divorced/separated	11	9.57
widow/widower	5	4.35
**5. Education**		
junior secondary school or lower	8	6.96
vocational	29	25.22
secondary education	43	37.39
higher education	35	30.43
**6. Professional status**		
Employed	21	18.26
Self-employed	16	13.91
Housewife	7	6.09
Retired	16	13.91
Professionally inactive because of health condition	45	39.13
Professionally inactive—other	10	8.70
**7. Duration of the disease since its diagnosis (in years)**	6.74	4.41
**8. Comorbidities**		
0	26	22.61
1	40	34.78
2	24	20.87
3	12	10.43
4 or more	13	11.00
**9. Depression diagnosed by a doctor**		
yes	90	78.26
no	25	21.74
10. BMI	23.16	3.31
**11. Adapted interior of the house to the needs**		
Definitely yes	36	31.30
Rather yes	45	39.13
Rather no	24	20.87
Definitely no	10	8.70
**12. Adaptation of the house environment to the needs**		
Definitely yes	27	23.48
Rather yes	37	32.17
Rather no	36	31.30
Definitely no	15	13.04
**13. Stages of the disease**		
Stage I (TFC 11–13 points)	35	30.43
Stage II (TFC 7–10 points)	33	28.70
Stage III (TFC 3–6 points)	25	21.74
Stage IV or V (TFC 1–2; 0 points)	22	19.13
**14. 12-item WHODAS 2.0 total score (0–60)**	36.54	15.45
Cognitive functions (0–10)	5.97	2.75
Mobility (0–10)	6.48	2.78
Self-care (0–10)	5.66	3.05
Getting along (0–10)	5.43	2.81
Life activities (0–10)	6.37	2.90
Participation in social life (0–10)	6.63	2.39
**15. Quality of life total score**	52.69	26.67
Motor functioning dimension	50.11	32.64
Psychology dimension	42.39	28.12
Socializing dimension	69.86	26.85

BMI, Body Mass Index; TFC, Total Functional Capacity; WHODAS 2.0, World Health Organization Disability Assessment Schedule 2.0.

**Table 2:** Scale score reliability of the 12-item WHODAS 2.0.

12-item WHODAS 2.0	Study Population *n* = 115		Test–Retest *n* = 20			
	Mean (SD)	Cronbach’s α	Test	Retest	*p*-Value	ICC
Total disability	36.54 (15.45)	0.966	38.55 (14.75)	39.20 (12.85)	0.230 ^a^	0.99
Do1. Cognition	5.97 (2.75)	0.956	6.25 (2.75)	6.35 (2.54)	0.529 ^b^	0.97
Do2. Mobility	6.48 (2.78)	0.956	7.00 (2.49)	7.20 (2.26)	0.441 ^b^	0.92
Do3. Self-care	5.66 (3.05)	0.962	6.40 (2.68)	6.40 (2.14)	0.859 ^b^	0.93
Do4. Getting along	5.43 (2.81)	0.965	5.40 (2.70)	5.55 (2.76)	0.311 ^b^	0.99
Do5. Life activities	6.37 (2.90)	0.954	6.80 (2.86)	6.70 (2.66)	0.463 ^b^	0.99
Do6. Participation	6.63 (2.39)	0.963	6.70 (2.23)	7.00 (1.84)	0.193 ^b^	0.91

^a^ Student’s *t*-test for dependent samples. ^b^ Wilcoxon test. WHODAS 2.0, World Health Organization Disability Assessment Schedule 2.0.

**Table 3:** The correlation of the 12-item WHODAS 2.0 and the H-QoL-I domains.

12-item WHODAS 2.0 H-QoL-I	Total Disability	Do1 Cognition	Do2 Mobility	Do3 Self-Care	Do4 Getting Along	Do5 Life Activities	Do6 Participation
**Quality of life total score**	−0.90	−0.83	−0.85	−0.83	−0.78	−0.87	−0.87
**Motor functioning dimension**	−0.89	−0.84	−0.86	−0.84	−0.75	−0.87	−0.84
**Psychology dimension**	−0.77	−0.71	−0.73	−0.75	−0.64	−0.73	−0.76
**Socializing dimension**	−0.79	−0.74	−0.77	−0.66	−0.76	−0.77	−0.76

All coefficients were statistically significant (*p* < 0.001). WHODAS 2.0, World Health Organization Disability Assessment Schedule; H-QoL-I, Huntington Quality of Life Instrument 2.0.

**Table 4:** Known-group validity of the 12-item WHODAS 2.0.

Groups		*n*	Mean	SD	Me	*p*-Value
**TFC**	I and II	68	26.06	10.10	24.50	<0.001 ^c^
	III and IV	47	51.70	6.78	53.00	

^c^ Mann–Whitney test. TFC, Total Functional Capacity.

**Table 5:** Impact of factors related to the level of disability.

Selected Factors	Disability			
	**B**	**(95 CI)**		***p*-Value**
Gender (reference male)	−1.21	−3.11	0.70	0.212
Age	0.01	−0.16	0.18	0.899
BMI (in points)	−0.83	−1.45	−0.20	0.010
Duration of the disease (in years)	1.39	0.89	1.90	<0.001
Marital status (reference in a relationship)	−0.17	−2.25	1.90	0.868
Place of residence (reference rural area)	2.78	0.78	4.77	0.007
Education (reference at least secondary education)	0.62	−1.46	2.71	0.555
Professional status (reference professionally active)	7.78	5.29	10.28	<0.001
Adaptation of interior of flat/house to the needs of everyday functioning (reference fully adapted)	0.22	−2.29	2.72	0.864
Adaptation of residential environment to the needs of everyday functioning (reference fully adapted)	−0.64	−3.13	1.86	0.614
Number of comorbidities	0.01	−1.40	1.42	0.989
Depression (reference no)	−0.69	−3.11	1.73	0.574

**Table 6:** Impact of factors related to the level of QoL.

Selected Factors	QoL			
	B	(95 CI)		*p*-Value
Gender (reference male)	3.84	0.49	7.19	0.025
Age	0.02	−0.27	0.32	0.875
BMI (in points)	1.46	0.36	2.56	0.010
Duration of the disease (in years)	−2.87	−3.75	−1.98	<0.001
Marital status (reference in a relationship)	1.03	−2.62	4.68	0.576
Place of residence (reference rural area)	−4.05	−7.56	−0.54	0.024
Education (reference at least secondary education)	−2.18	−5.84	1.49	0.242
Professional status (reference professionally active)	−9.70	−14.09	−5.31	<0.001
Adaptation of interior of flat/house to the needs of everyday functioning (reference fully adapted)	−1.36	−5.76	3.03	0.540
Adaptation of residential environment to the needs of everyday functioning (reference fully adapted)	0.02	−4.36	4.39	0.994
Number of comorbidities	−0.92	−3.40	1.56	0.463
Depression (reference no)	1.32	−2.94	5.58	0.539

QoL, Quality of Life; BMI, Body Mass Index.

## Supplementary Materials

The following are available online at https://www.mdpi.com/2077-0383/10/5/1053/s1, Table S1: Correlations between individual domains and the overall result (*n* = 115); Table S2: Correlations between the WHODAS 2.0 version 12 items and the overall score and domains.
