# Supplementary material for: Psychometric Properties and Validation of the Polish Version of the 12-Item World Health Organization Disability Assessment Schedule 2.0 in Patients with Huntington’s Disease
Source: J Clin Med. 2021 Mar 4;10(5):1053. doi: 10.3390/jcm10051053 (PMC7961505; doi:10.3390/jcm10051053)
Supplement: Supplementary file 1 [file jcm-10-01053-s001.pdf]

Journal of
Clinical Medicine

Table S1. Correlations between individual domains and the overall result (n = 115).

WHODAS 2.0 version 12	Total disability	Do1 Cognition	Do2 Mobility	Do3 Self-care	Do4 Getting along	Do5 Life activities	Do6 Participati on
Total disability	1.00	0.94	0.94	0.91	0.88	0.95	0.90
Do1 Cognition	0.94	1.00	0.89	0.87	0.81	0.88	0.81
Do2 Mobility	0.94	0.89	1.00	0.86	0.79	0.89	0.83
Do3 Self-care	0.91	0.87	0.86	1.00	0.72	0.86	0.76
Do4 Getting along	0.88	0.81	0.79	0.72	1.00	0.82	0.79
Do5 Life activities	0.95	0.88	0.89	0.86	0.82	1.00	0.87
Do6 Participation	0.90	0.81	0.83	0.76	0.79	0.87	1.00

All coefficients were statistically significant ($p < 0.001$)

WHODAS 2.0, World Health Organization Disability Assessment Schedule

Table S2. Correlations between the WHODAS 2.0 version 12 items and the overall score and domains.

Domain components of WHODAS 2.0 version 12	Domains	Overall score
	**Do1 Cognition**	
Do1 - S3	0.94	0.89
Do1 - S6	0.92	0.86
	**Do2 Mobility**	
Do2 – S1	0.95	0.89
Do2 – S7	0.96	0.90
	**Do3 Self-care**	
Do3 – S8	0.98	0.90
Do3 – S9	0.98	0.89
	**Do4 Getting along**	
Do4 – S10	0.96	0.88
Do4 – S11	0.95	0.82
	**Do5 Life activities**	
Do5 – S2	0.97	0.95
Do5 – S12	0.97	0.91
	**Do6 Participation**	
Do6 – S4	0.95	0.92
Do6 – S5	0.82	0.62

All coefficients were statistically significant ($p < 0.001$)

WHODAS 2.0, World Health Organization Disability Assessment Schedule
